# Left Ventricular Function and Iron Loading Status in a Tertiary Center Hemochromatosis Cohort—A Cardiac Magnetic Resonance Study

**DOI:** 10.3390/diagnostics12112620

**Published:** 2022-10-28

**Authors:** Karolina Dorniak, Ludmiła Daniłowicz-Szymanowicz, Katarzyna Sikorska, Katarzyna Rozwadowska, Jadwiga Fijałkowska, Anna Glińska, Magdalena Tuzimek, Agnieszka Sabisz, Marta Żarczyńska-Buchowiecka, Michał Świątczak, Maria Dudziak, Edyta Szurowska

**Affiliations:** 1 Department of Noninvasive Cardiac Diagnostics, Medical University of Gdansk, Dębinki 7, 80-210 Gdansk, Poland; 2 Department of Cardiology and Electrotherapy, Medical University of Gdansk, Dębinki 7, 80-210 Gdansk, Poland; 3 Department of Tropical Medicine and Infectious Diseases, Medical University of Gdansk, Dębinki 7, 80-210 Gdansk, Poland; 4 2nd Department of Radiology, Medical University of Gdansk, Dębinki 7, 80-210 Gdansk, Poland

**Keywords:** hemochromatosis, cardiac magnetic resonance, T2* mapping

## Abstract

Background: Haemochromatosis (HCH), a common genetic disorder with variable penetrance, results in progressive but understudied iron overload. We prospectively evaluated organ iron loading and cardiac function in a tertiary center HCH cohort. Methods: 42 HCH patients (47 ± 14 years) and 36 controls underwent laboratory workup and cardiac magnetic resonance (CMR), including T1 and T2* mapping. Results: Myocardial T2* (myoT2*), myocardial T1 (myoT1) and liver T2* (livT2*) were lower in patients compared to controls (33 ± 4 ms vs. 36 ± 3 ms [*p* = 0.004], 964 ± 33 ms vs. 979 ± 25 ms [*p* = 0.028] and 21 ± 10 ms vs. 30 ± 5 ms [*p* < 0.001], respectively). MyoT2* did not reach the threshold of clinically significant iron overload (<20 ms), in any of the patients. In 22 (52.4%) patients, at least one of the tissue parameters was reduced. Reduced myocardial T2* and/or T1 were found in 10 (23.8%) patients, including 4 pts with normal livT2*. LivT2* was reduced in 18 (42.9%) patients. MyoT1 and livT2* inversely correlated with ferritin (rs = −0.351 [*p* = 0.028] and rs = −0.602 [*p* < 0.001], respectively). LivT2* by a dedicated sequence and livT2* by cardiac T2* mapping showed good agreement (ICC = 0.876 *p* < 0.001). Conclusions: In contemporary hemochromatosis, significant myocardial iron overload is rare. Low myocardial T2* and/or T1 values may warrant closer follow-up for accelerated myocardial iron overload even in patients without overt liver overload. Cardiac T2* mapping sequence allows for liver screening at the time of CMR.

## 1. Introduction

Hemochromatosis (HCH), a multisystemic disease resulting from iron overload, is one of the most common genetic disorders in Caucasians; 1–2 per 500 people are affected by one of the several disease types [1,2]. Homozygous mutations in the HFE gene (C282Y/C282Y) are responsible for most cases. Other HFE gene mutations and mutations in other genes, the products of which play a role in the iron turnover pathways, are occasionally involved [3,4,5]. The phenotypic penetrance of HCH-related mutations varies. The laboratory penetrance, with increased iron turnover parameters such as ferritin and transferrin saturation, is high at 38–76% [6]. The clinical penetrance was demonstrated to be lower and gender-dependent, with higher rates in men (2–38%) and lower rates in women (1–10%) [7,8,9]. Host-related factors such as alcohol, inflammation, and viral infections seem to precipitate clinical penetrance [10,11]. 

Oxidative stress resulting from an accumulation of bioactive iron leads to tissue damage and organ dysfunction [12,13]. Most commonly, the liver is the primary target organ for iron overload, with a high risk of disease progression and the development of cirrhosis and hepatocellular carcinoma in patients who are not properly treated [14]. The skin and endocrine tissues may be involved, such as pancreatic islets and the thyroid, where the damage can become irreversible at earlier stages [15,16]. Patients are also at risk of developing bone architectural disorders and arthropathy [17,18]. Cardiomyocytes, due to their intense metabolism, are particularly susceptible to the toxic effects of iron. Hence, myocardial iron accumulation could lead to cardiomyopathy and heart failure [19], which represents a relatively late but potentially fatal manifestation of HCH. Heart failure and hepatic carcinoma are the well-known leading causes of death in HCH patients, with approximately one-third dying of cardiac causes [15,16]. Nowadays, since genetic testing can be easily performed, the disease is more likely to be diagnosed at the early stages. This allows for the timely initiation of treatment to prevent organ damage, including symptomatic heart involvement [20,21,22,23].

Echocardiography is an established noninvasive tool for cardiomyopathies, irrespective of their etiology. However, it has poor sensitivity for early myocardial involvement in HCH when standard techniques are used. More advanced techniques such as two-dimensional speckle tracking echocardiography (2D STE) have shown potential for early detection of myocardial alterations in HCH patients [24,25,26]. 

Liver iron loading has been traditionally assessed by liver biopsy [14,26]. The steadily increasing availability of quantitative magnetic resonance techniques for iron assessment, however, such as multi-echo gradient-echo sequence, promoted the noninvasive approach. Similar sequences, adapted to myocardial tissue characteristics, can also be utilized for myocardial iron and have been proven extremely effective in β-thalassemia major, leading to a spectacular decrease in cardiovascular mortality rates [27]. They have been utilized in other iron overload disorders, including hereditary hemochromatosis, sickle cell disease, and other transfusion-dependent conditions [28,29,30,31]. These MR techniques have been validated against biopsy in both the heart and the liver [32,33].

Myocardial T2* decreases as iron accumulates, with the normal myocardial T2* being approximately 35–40 ms. T2* values of <20 ms reflect significant myocardial iron overload [34], and values below 10 ms denote a high risk of developing heart failure [35]. With recent cardiac magnetic resonance (CMR) advancements, pixel-wise measurement has become possible with cardiac T2* mapping, which has been demonstrated to be an accurate and reliable tool for the quantification of cardiac iron [28]. Moreover, T2* mapping has been shown to be effective in therapy monitoring [36,37]. Recently, another parametric technique that measures myocardial longitudinal relaxation time T1 (T1 mapping) has been proven sensitive to cardiac iron overload [38,39], showing better reproducibility when compared to T2* mapping [36]. Quantitative CMR with parametric mapping for iron overload was employed in many studies, but not specifically in the setting of genetically confirmed HCH [40,41,42,43].

Given the availability of appropriate noninvasive tools, myocardial assessment in the earlier stages of HCH, before significant damage to the LV is evident, seems achievable and may have significant diagnostic and prognostic implications. In this CMR study, we aimed to evaluate myocardial and liver iron status in a contemporary HCH cohort at a tertiary center and to compare these values in the treatment of naive and treated patients. We also assess the feasibility of cardiac parametric mapping for liver overload assessment during CMR. Additionally, inter-observer variability of the T2* mapping was evaluated.

## 2. Materials and Methods

### 2.1. Study Population

Forty-two patients of Caucasian descent aged 47 ± 14 years (13 [31%] female), with HCH without cardiovascular symptoms, were prospectively included between November 2014 and January 2019. The disease was diagnosed based on the presence of elevated serum iron parameters in the blood and the detection of the homozygous C282Y/C282Y HFE gene mutation. In carriers of other HFE genotypes (mixed heterozygotes C282Y/H63D or H63D/H63D homozygotes), excessive iron accumulation in hepatocytes was confirmed by a histopathological examination of the liver biopsy specimen or MR imaging. Other reasons for iron loading (alcohol or metabolic dysfunction associated with fatty liver disease, and iron-loading anemias) were excluded [44]. The laboratory workup included: iron, serum ferritin, transferrin saturation (TSAT), hemoglobin, liver function tests, and glucose levels. The exclusion criteria included: age < 18 years; absolute contraindications to MR imaging; ongoing symptoms of cardiovascular disease; and echocardiographic LV ejection fraction (LVEF) < 50%.

Patients were divided into two subgroups: Treatment-naive (0–1 venesections, n = 27; median 6 [1–15] months from diagnosis) and on treatment (two or more venesections, n = 15; median 128 [56–180] months from diagnosis); treatment according to clinical practice recommendations (14). The control group included 36 healthy age- and gender-matched volunteers. The study protocol was approved by the Institutional Ethics Committee (NKBBN/452/2016), and written informed consent was obtained from all subjects.

### 2.2. CMR Image Acquisition

ECG-gated CMR examinations were performed using a 1.5 T MR scanner (Siemens Magnetom Aera, Erlangen, Germany) with an 18-element phased array cardiac coil and a 32-element spine coil with an auto-coil selection option, in line with SCMR/EuroCMR recommendations [45]. T1 and T2* mapping sequences (MyoMaps, Siemens, Erlangen, Germany) based on modified Look-Locker inversion recovery (MOLLI) and multi-echo gradient-echo sequences, respectively, were prescribed in a short-axis view (including liver tissue in the field of view). For myocardial T2*, typical acquisition parameters were as follows: min echo time: 2 ms; echo time interval 2 ms; the number of echoes: 8 (from 2.1 to 16.2 ms); repetition time depending on heart rate, flip angle 20–35°; bandwidth 810 Hz per pixel, iPAT 2. The T1 relaxation time was mapped with the MOLLI sequence: echo time 1.1 ms; the number of TI: 8 (from 100 ms to 2500 ms); repetition time depending on heart rate; flip angle 35 o, bandwidth 1080 Hz per pixel, iPAT 2. The field of view was adapted to each patient, but the average voxel size was 1.6 x 1.6 mm, and the average slice thickness was 8 mm. For liver T2*, a dedicated multi-echo gradient-echo sequence was performed in an axial plane, with fourteen TEs (from 1.15 to 17.01 ms) and a fixed TR of 200 ms, FOV of 450mm, slice thickness of 5mm, and an axial orientation voxel size of 3.5 × 3.5 mm.

These sequences were added to the routine protocol, which included cine and LGE short-axis CMR images prescribed every 10 mm (slice thickness of 8 mm) from base to apex. The in-plane resolution was typically 1.4 × 1.4 mm. Cine CMR was performed using a steady-state-free precession sequence. LGE images were acquired on average 8–15 min after contrast administration using segmented IR-GRE with inversion time adjusted as needed. The contrast (gadobutrol) dose was 0.1 mmol/kg.

### 2.3. CMR Image Analysis

A dedicated module of the software Segment (Medviso AB, Lund, Sweden) for magnitude data analysis was used, which included error maps, showing pixel-wise fitting error levels. All parametric maps were generated by this software and then evaluated visually. Only areas with a fitting error level of 5% or less were analyzed, and preselected ROI placement was adjusted to meet this requirement to ensure the high quality of the data [46]. An optimum contrast TE image was used for ROI placement in the septum.

For myocardial T2* measurement, a full-thickness region of interest (ROI) was measured in the septum to avoid artifacts related to the proximity of the cardiac veins and lungs. For T1 measurement using a modified Look-Locker inversion recovery (MOLLI) sequence, a similar ROI was evaluated for comparisons and checked with LGE images to avoid areas of fibrosis/scar. An ROI of no less than 2 cm^2^ away from any visible vessels was analyzed in the liver. 

Values outside the reference range (RR, defined as mean ± 1.96 SD: 30–42 ms) based on the healthy control group, i.e., myoT2* below 30 ms, myoT1 below 928 ms (RR: 928–1028 ms), and liv T2* below 20 (RR: 20–40 ms) were defined as abnormal. Left ventricular volumes and ejection fraction were calculated based on semi-automatic endocardial and epicardial contours placed on the short-axis cine in the end-systolic and end-diastolic phases (SyngoVia VB30, Siemens Healthineers, Erlangen, Germany). The LV mass was calculated by subtracting the endocardial from the epicardial volume at end-diastole and multiplying by 1.05 g/cm^3^. The LGE was assessed visually by two observers, and any discrepancy was solved by consensus with the 5SD method [47].

Two observers (10 years and one year of experience in CMR analysis) blinded to other patient information and each other’s results performed independent analyses in a subset of subjects to calculate inter-observer variability (reproducibility) of myocardial T2*. Moreover, the level of concordance between liver T2* by cardiac mapping and the routine dedicated gradient-echo multi-echo sequence was assessed. The data presented, except for comparisons between observers, are based on measurements by the observer with 10 years of experience.

### 2.4. Statistics

Continuous data are expressed as mean (SD) or median (25th–75th percentiles) and categorical data are expressed as proportions. The Shapiro–Wilk test was used to determine whether the data were normally distributed. Whenever the variables did not display normal data distributions, nonparametric tests were used. Comparisons between groups were performed with the Mann–Whitney U test for continuous variables and Pearson’s chi-square test for categorical variables, as appropriate. The correlations between iron turnover and the MR relaxometry parameters were evaluated using Spearman’s correlation coefficient. Intraclass correlation coefficient and Bland–Altman plots were performed for interobserver variability analysis between experienced and less experienced observers as well as for the liver T2* measurement concordance between the cardiac mapping and dedicated multi-echo sequence; *p*-values < 0.05 were considered significant. The statistical analysis was conducted with IBM SPSS Statistics Software v25. 

## 3. Results

The homozygous C282Y/C282Y genotype was found in 30 (71.4%) patients, with the compound heterozygous C282Y/H63D genotype found in seven patients (16.7%), C282Y/wt was found in one (2.4%), and H63D/H63D was found in four (9.5%). The mean age of the patients was 47 ± 14 years, with male predominance (69%). Twelve of these patients had diabetes or impaired fasting glucose levels.

All left ventricular (LV) volume and function parameters were within normal ranges [48], with no differences between HCH patients and controls. Similarly, no differences were found between subgroups (treatment-naive vs. on-treatment). The demographic and clinical characteristics of the study subjects are presented in Table 1.

Table 2 shows the iron turnover and liver function parameters of the patients, as well as their hemoglobin and glucose levels at the time of enrollment.

**Figure 1:**
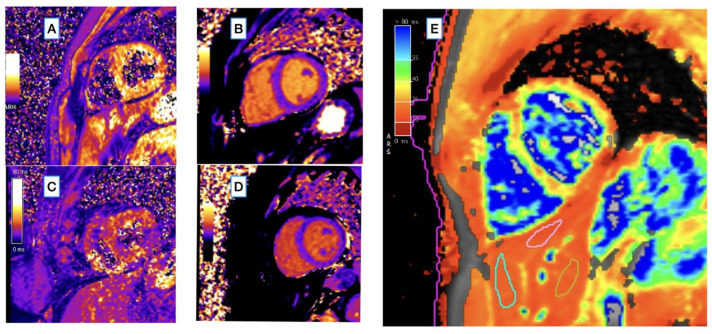
(**A**–**D**) Inline T1 and T2* maps generated with MyoMaps (Siemens Healthineers, Erlangen, Germany) showing myocardial T2* and myocardial T1 maps in healthy control ((**A**) average T2* = 35 ms, and (**B**) average T1 = 962 ms) and in an HCH patient with reduced myocardial T2* and normal T1 relaxation times ((**C**) average T2* = 23 ms, and (**D**) average T1 = 988 ms). The institutional reference ranges of T1 and T2* were 993 ± 21 ms [951–1035 ms] and 36.5 ± 3.8 ms [29–44 ms], respectively. (**E**) An example of ROI placement for liver T2* measurement in a hemochromatosis patient using the cardiac T2* mapping sequence (average liver T2* in the three ROIs shown in the right panel was 14.9 ms, which corresponds to 1.67 mg [Fe] per gram dry weight). This map was generated with cvi42 (Circle Cardiovascular Imaging, Calgary, Canada). MOLLI, modified Look-Locker inversion recovery; SAX, short axis.

Both myocardial T2* (myoT2*) and myocardial T1 (myoT1) as well as liver T2* (livT2*) were significantly lower in HCH patients compared with controls (Figure 1). In none of the HCH patients did myoT2* values reach a value of below 20 ms (an established threshold for clinically significant iron overload [49]. Median myocardial T2* and T1 values in HCH patients were significantly lower than in controls (34 [32–36] ms vs. 36 [34–38] ms, *p* = 0.004 and 962 [947–988] ms vs. 981 [961–998] ms, *p* = 0.028). 

Median liver T2* by cardiac mapping was also lower than in controls (21 [17–32] ms vs. 31 [28–33] ms, *p* = 0.004). Myocardial and hepatic relaxometry parameters are summarized in Table 3.

As shown in Table 4, abnormal values (i.e., below the reference range) of one or more relaxometry parameters of the liver and/or myocardium were found in 22 (52.4%) HCH patients (15 [68%] untreated). MyoT2* and/or myoT1 value below RR were found in 10 (23.8%) HCH pts. LivT2* fell below the reference range in 18 (42.9%) HCH patients. 

No significant changes were noted between treatment subgroups concerning CMR-derived organ iron loading parameters (Table 5). In six patients, small areas of LGE were noted (ischemic type in two and non-ischemic type in four).

No correlations were found between LVEDVI and myoT2* (*p* = 0.275). Similarly, no correlation was found between ferritin and myoT2* (rs = −0.125; *p* = 0.442). Conversely, myoT1 values as well as livT2* showed a rather weak but significant inverse correlation with ferritin concentration (rs = −0.351 [*p* = 0.028] and rs = −0.602 [*p* < 0.001], respectively). Of note, there was no correlation between myoT1 and myoT2* (rs = 0.199; *p* = 0.087).

**Figure 2:**
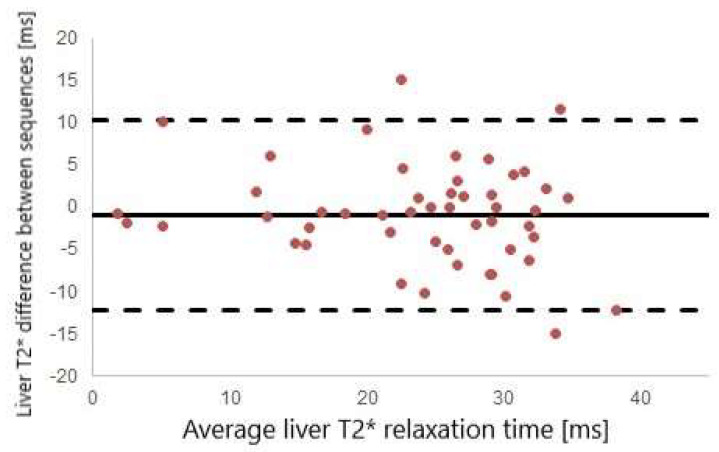
Bland–Altman plot of liver T2* values measured using a dedicated multi-echo gradient echo sequence and cardiac T2*map.

**Figure 3:**
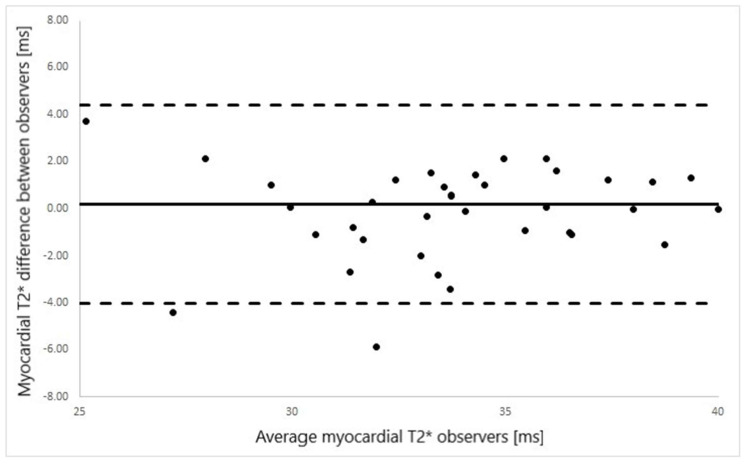
Bland–Altman plot of myocardial T2* values measured by two observers (9 years and 1 year of CMR experience) from the cardiac T2*map.

Good agreement was found between liver T2* values as measured by standard gradient-echo multi-echo sequence and by cardiac T2* map (ICC = 0.876 *p* < 0.001, Figure 2). Similarly, good interobserver agreement was found for myoT2* values (ICC = 0.94; *p* < 0.001, Figure 3). 

## 4. Discussion

Given the relative paucity of studies on organ iron loading in hemochromatosis, this study offers important new insights. In our tertiary center cohort of HCH patients without symptoms of cardiovascular disease, standard LV volume and function parameters were not useful for iron overload risk assessment as they did not differ from healthy controls. Conversely, the CMR-derived iron loading parameters (i.e., myocardial T2*, myocardial T1, and liver T2*) were significantly lower than in healthy controls. Of note, one or more of these parameters were below the reference range in over 50% of the study group. Low liver T2* (livT2*) was by far the most common abnormality (18 [42%] pts, of which 13 [72%] were untreated), with features of significant liver iron loading (i.e., livT2* below 10 ms as assessed by the MyoMaps T2* sequence) found in only 6 pts (10%). Ten patients (24%) showed abnormally low myocardial relaxation parameters (myoT1, myoT2*, or both, below the reference range). Hence, although myocardial iron overload was not clinically significant in any of the patients included in the study, it is likely that the pathological process may already be ongoing in a proportion of recently diagnosed treatment-naive individuals, while it is sufficiently controlled in the treated subgroup. This, given the high prevalence and variable clinical penetrance of the HFE gene mutations, suggests the need for early risk stratification and the potential for tailored preventive strategies. 

Importantly, the subgroup with low myocardial T1 and/or T2* included four patients with normal liver T2*, three of which were recently diagnosed, as treatment-naive patients. This points out the possibility that, in the presence of environmental or host-related modifiers, myocardial iron accumulation may occur in parallel or even precede significant hepatic overload [1]. In line with a previous report suggesting that significant cardiac involvement at the early stages of HFE-HCH is a rare finding, none of the untreated patients with recently diagnosed HCH included in our study had clinically significant myocardial iron overload as measured by T2* [50]. Of note, the myoT2* and myoT1 values in our control group were similar to previously published reports [40,51].

Low myocardial T1 (myoT1) has been found to be a useful indicator of iron overload in various settings, including thalassemias, other transfusion-dependent anemias, hemochromatosis, and myelodysplastic syndromes [39,40,51]. It showed better sensitivity and less measurement variability compared to T2*, particularly in milder diseases [40]. However, as opposed to T2*, no clear and widely accepted cutoff values beyond the healthy reference range exist for iron loading measured by T1. In a study on T1 in thalassemic patients, Torlasco and co-workers proposed a T1 cutoff value of 918 ms, representing a lower limit of 95% CI for healthy volunteers, which is similar to the range adopted in our study. Krittayaphong et al. proposed a cutoff native T1 value of 900 msec in thalassemia patients with cardiac iron overload [52]. However, this cutoff value was not derived from healthy controls but from patients with no iron overload as defined by myoT2* > 20 ms. Importantly, such range limits can only be applicable in the setting of the same technique and field strength (i.e., modified Look-Locker inversion recovery sequence [MOLLI] at 1.5T) and, ideally, they should be determined for each MR scanner. In our study, similar to previously published findings by Sado [40] and Torlasco [52], myocardial T1 values were markedly lower in patients (both in the treated and untreated subgroups) than in controls. However, in these studies, T1 was found to either correlate with T2* [40] or displayed a more complex, nonlinear relationship with T2* [52] as opposed to our data. We found no correlation between cardiac T1 and T2*. This may be partially due to T1 being potentially affected by several factors independent of iron accumulation. Namely, patients in our study were a decade older and thus had a higher probability of subclinical myocardial changes, which would typically promote T1 increase, thus counteracting T1 lowering related to iron loading [30]. Moreover, both T1 and T2* values in the study by Sado were generally lower compared to our patients, implicating more advanced iron loading in a non-homogenous patient group (predominantly non-HCH) [40]. Of note, no patient with low T2* in their study had normal T1, as opposed to our work where two patients had T2* below the reference range with T1 within the normal range. As previously mentioned, this may be related to the overlapping subclinical myocardial disease (unrelated to iron accumulation) and the promoting myocardial T1 increase. It seems likely, given that both patients were in their sixth decade of life and both non-CAD-type late gadolinium enhancement were present, suggestive of earlier subclinical events likely unrelated to HCH, such as myocarditis. Conversely, a proportion of our patients had low myocardial T1 and normal T2*, which is in line with several previously published reports [39,40,51]. Sado et al. reported low myocardial T1 values in as many as 33% of iron-loaded patients with T2* > 20 ms [40], and similar results were reported by Torlasco [52]. Given the fact that in the setting of low global native myocardial T1, the sole differential to cardiac iron overload is Anderson-Fabry disease (glycosphingolipid storage disease due to alpha-galactosidase A deficiency) and that the overlap between these entities is unlikely, native myocardial T1-mapping emerges as an important tool for myocardial iron overload detection. 

It should also be noted that myoT1 values in our cohort correlated significantly with ferritin values, as opposed to myoT2* values showing weak or no correlations with iron turnover parameters (which is concordant with a previously published study on HCH patients) [31]. In this study, however, in a similar HCH cohort, up to 20% of subjects displayed significant myocardial iron loading (as opposed to our results where no cases of significant myocardial iron overload were identified), which might be at least in part due to the fact that these patients were over a decade older than our group, and the study itself was published almost a decade earlier [31]. One should also bear in mind that the phenotypic penetrance of HCH has well-documented ethnic variability, which may preclude direct comparisons between HCH cohorts across different nationalities and/or regions [53]. 

Patients with myoT2* values > 20 ms are unlikely to have significant iron overload at the time of scanning, but those with borderline T2* and/or low T1 values might still deserve attention as a proportion of these patients will likely develop iron-overload cardiomyopathy in the future [1]. Consequently, for an effective screening and prevention strategy, iron overload should most likely be regarded as a continuum rather than a binary entity. Given the highly variable phenotypic penetrance, stratifying risk at the time of diagnosis and adapting the pace of further screening to the initial findings seems a reasonable approach in hereditary hemochromatosis. However, further longitudinal outcome studies are required to develop an informed strategy in this setting.

Limited availability of CMR requires efficient use of scanner time, and measures should be taken to optimize exam protocols. Accordingly, we have shown that in patients with HCH, liver T2* values derived from the cardiac T2* mapping sequence did not differ from values acquired in a dedicated sequence with a broader range of echo times that represents a standard approach for liver assessment. It should be underlined that for robust T2* measurements in a wide range of T2* values, both liver and myocardial T2* times were measured with the combined signal model algorithm incorporated in the software Segment [54]. Thus, cardiac T2* mapping can be used for both myocardial and liver T2* measurement, and a separate dedicated multi-echo gradient-echo sequence for liver assessment can be used optionally, specifically when cardiac T2* maps suggest markedly reduced liver T2* values. The use of parametric mapping is also in line with a recent review of T2* measurement methods, where T2* measurement that involves pixel-wise quantification is considered the state of the art as it provides better spatial iron loading information compared with quantification based on a region of interest [29]. Thus, cardiac MR with T2* mapping could potentially eliminate the need for targeted liver scanning in a proportion of patients, e.g., those with normal or nearly normal liver T2* values. Of note, the interobserver variability of T2* measurements in our study is similar to the results that have been reported previously [31], demonstrating that the technique is a reliable tool irrespective of the observer’s experience.

## 5. Study Limitations

Our study has some limitations. Firstly, a relatively low number of subjects were included. However, this resulted from the strict inclusion criteria that included genetic testing, while additional genetic testing for rare mutations in HFE and other iron-related genes was not performed. Secondly, interobserver variability was assessed in a subgroup of our patients. Lastly, excess iron deposition begins within the epicardium and extends towards the mid-myocardium and endocardium. This was not specifically addressed in our methodology as best intramyocardial ROIs were selected to avoid partial volume effect and hence, the results may have been confounded by the degree of transmurality of iron deposits. Further research with a larger sample size and a more complex methodology could help to address this issue.

## 6. Conclusions

In patients with hemochromatosis, standard LV volume and function parameters are not helpful for myocardial iron loading screening, irrespective of the time from diagnosis and the treatment status. Significant myocardial iron overload (i.e., myocardial T2* below 20 ms) is a rare finding in a contemporary HCH cohort at a tertiary center. However, abnormally low T2* and T1 values at the time of diagnosis could help identify patients at higher risk of cardiac iron overload even in the absence of liver overload and may warrant closer follow-up. Further research in larger cohorts is needed to demonstrate the clinical benefits of such an approach. Liver T2* measurement based on the cardiac T2* mapping sequence at the time of CMR can be useful for simultaneous liver screening.

## Figures and Tables

**Table 1:** Demographic and clinical characteristics of the study group. Data are presented as median (Q1–Q3).

	Patients (N = 42)	Controls (N = 36)	*p* Value
Age [years] median (Q1–Q3)	47 (36–59)	42 (29–55)	0.140
Sex (F)	13	16	0.219
LVEF median (Q1–Q3)	81 (70–92)	70 (58–82)	0.211
LVEDVI median (Q1–Q3)	32 (25–39)	31 (23–39)	0.691
LVESVI median (Q1–Q3)	67 (58–76)	62 (53–71)	0.551
LVMI median (Q1–Q3)	60 (56–64)	61 (57–65)	0.148

**Table 2:** Iron turnover parameters in patients. Data are presented as median (25th–75th percentile).

Parameter	Median (Q1–Q3)	Range (Min–Max)
Iron (μg/dL)	204 (173–235)	62–283
Ferritin (ng/mL)	625 (238–1012)	100–3550
TSAT [%]	82 (67–97)	48–100
Haemoglobin (mg/dL)	15 (14.1–15.9)	12.0–17.8
Glycemia (mg %)	96 (85–107)	75–139
AsT	26 (20–38.3)	13–76
AlaT	35 (23.5–46.5)	11–150

**Table 3:** Relaxometry parameters in the study group. Data are presented as median (25th–75th percentile). MyoT1—myocardial T1 relaxation time constant; myoT2*—myocardial T2* relaxation time constant; livT2*—liver T2* relaxation time constant. GRE—gradient echo sequence MyoMaps—A set of T1, T2, T2* mapping sequences, Siemens Healthineers, Erlangen, Germany.

Parameter	Patients	Controls	*p*
myoT2* [ms] (MyoMaps) median (Q1–Q3)	34 (32–36)	36 (34–38)	*p* = 0.004
myoT1 [ms] (MyoMaps) median (Q1–Q3)	962 (947–988)	981 (961–998)	*p* = 0.028
livT2* [ms] (MyoMaps) median (Q1–Q3)	23 (17–30)	31 (28–38)	*p* = 0.004
livT2* [ms] (multiecho GRE) median (Q1–Q3)	25 (17–30)	27 (23–34)	*p* = 0.041

**Table 4:** The prevalence of abnormally low relaxometry parameters (myoT2*, myoT1, and/or livT2*) in the study group. Abbreviations as in Table 3.

Relaxometry Parameter below the Reference Range	All N (%)	Treatment Naive N (%)	On Treatment N (%)
Single parameter	15 (35.7)	11 (73)	4 (27)
Low livT2*	12 (28.6)	9 (75)	3 (25)
Low myoT2*	1 (2.4)	0	1 (100)
Low myoT1	2 (4.7)	2 (100)	0
Multiple parameters	7 (16.7)	4 (57)	3 (43)
Low myoT2* and myoT1 + normal livT2*	1 (2.4)	1 (100)	0
Low myoT2* and myoT1 + low livT2*	3 (7.1)	2 (66.7)	1 (33.3)
Low myoT2* or low myoT1 + low livT2*	3 (7.1)	1 (33.3)	2 (66.7)
Total	22 (52.4)	15 (68.2)	7 (31.8)

**Table 5:** Iron loading parameters on CMR and laboratory tests (iron turnover, glycemia, and liver function) in patients by subgroup.

Treatment Status	On Treatment n = 24 (57%)	Treatment Naive n = 18 (43%)	*p*
Age [years] mean ± SD (range)	42 ± 15 (range 18–77)	51 ± 14 (range 20–68)	0.055
Gender, N	(F = 9, M = 15)	(F = 4, M = 14)	0.289
myoT2* [ms] median (Q1–Q3)	34 (32–37)	33 (31–35)	0.180
myoT1 [ms] median (Q1–Q3)	970 (946–988)	956 (951–973)	0.570
livT2* [ms] median (Q1–Q3)	20 (2–44)	22 (3–44)	0.434
Iron [mcg/dL] median (Q1–Q3)	196 (165–225)	206 (179–252)	0.286
Ferritin [mcg/dL] median (Q1–Q3)	421 (266–936)	714 (368–1037)	0.315
TSAT [mcg/dL] median (Q1–Q3)	81 (65–92)	84 (66–95)	0.447
Haemoglobin [mcg/dL] median (Q1–Q3)	15 (14.0–16.4)	15.3 (14.7–16.0)	0.716
Glucose [mcg/dL] median (Q1–Q3)	93 (85–102)	97 (89–111)	0.332
ASPAT [mcg/dL] median (Q1–Q3)	30 (20–39)	34 (24–53)	0.253
ALAT [mcg/dL] median (Q1–Q3)	39 (24–67)	55 (34–95)	0.143

## Data Availability

Data are available from the corresponding author upon reasonable request.
